# A Review on Machine Learning Applications for Solar Plants

**DOI:** 10.3390/s22239060

**Published:** 2022-11-22

**Authors:** Ekaterina Engel, Nikita Engel

**Affiliations:** Engineering Technological Institute, Katanov State University of Khakassia, Abakan 655017, Russia

**Keywords:** machine learning, neural networks, DL, PV, solar plant, smart sensor

## Abstract

A solar plant system has complex nonlinear dynamics with uncertainties due to variations in system parameters and insolation. Thereby, it is difficult to approximate these complex dynamics with conventional algorithms whereas Machine Learning (ML) methods yield the essential performance required. ML models are key units in recent sensor systems for solar plant design, forecasting, maintenance, and control to provide the best safety, reliability, robustness, and performance as compared to classical methods which are usually employed in the hardware and software of solar plants. Considering this, the goal of our paper is to explore and analyze ML technologies and their advantages and shortcomings as compared to classical methods for the design, forecasting, maintenance, and control of solar plants. In contrast with other review articles, our research briefly summarizes our intelligent, self-adaptive models for sizing, forecasting, maintenance, and control of a solar plant; sets benchmarks for performance comparison of the reviewed ML models for a solar plant’s system; proposes a simple but effective integration scheme of an ML sensor solar plant system’s implementation and outlines its future digital transformation into a smart solar plant based on the integrated cutting-edge technologies; and estimates the impact of ML technologies based on the proposed scheme on a solar plant value chain.

## 1. Introduction

Solar plant systems have complex nonlinear dynamics with uncertainties since the system’s parameters and insolation fluctuate [1]. Thereby, it is complicated to approximate these complex dynamics with classical methods, while ML methods provide the required performance [2]. In modern sensor systems, ML methods are crucial units to increase the quality of big dataset processing for solar plant design, forecasting, maintenance, and control [1,2]. Within the EU COVID-19 strategic reply, the smart energy standards define a cloud platform specification for a distributed solar big data ecosystem that will provide the creation of effective ML technologies for smart solar energy [3]. The long-term contribution of solar energy is dependent on overcoming the remaining issues of grid integration, high costs, and low efficiency, mainly through the research and development of a smart solar plant system based on ML methods on account of traditional methods’ ineffectiveness. Within breakthrough studies, ML technologies collected, analyzed, and converted a huge number of sensory datasets into ML knowledge. These big data sets are collected by supervisory control and data acquisition (SCADA) systems [4]. The SCADA system is able to integrate the sensor system and ML technologies into an ML sensor system based on software that implements ML sensor models and integrates with SCADA through API. Further, the application of ML technologies for the digital transformation of solar plant systems has a massive potential to increase their stability, reliability, dynamic response, cost-effectiveness, and other essential advancements, easing their integration into electric grids.

The contribution of this article is threefold. First, we reviewed more than 100 research papers devoted to state-of-the-art ML technologies of solar plant systems, most of the articles were published in the last five years. Second, we reviewed resources where researchers can find open datasets, source code, and ML framework and simulation environments to create ML technologies for a solar plant system. Third, in contrast with other review articles, our review proposes a simple but effective pipeline scheme for an ML sensor solar plant system’s implementation and outlines its future digital transformation into a smart solar plant based on integrated, cutting-edge technologies; estimates the impact of the ML technologies based on the proposed scheme on a solar plant value chain; sets benchmarks for performance comparison of the reviewed ML models for a solar plant’s system based on the comparative studies’ results summaries; and briefly summarizes our self-adaptive models for sizing, forecasting, maintenance, and control of a solar plant based on a modified fuzzy neural net (MFNN) that is automatically created with regard to tasks’ complexities and overfitting problems [5,6,7,8]. Our research provides a mapping of the recently reported ML methods and quantification of their advantage and shortcomings as compared to classical methods, which are usually employed in the sensor system, hardware, and software of solar plants; an effective integration scheme of ML technologies into the sensor systems and software of solar plants; a future prospect of the integrated cutting-edge technologies, including ML for digital transformation of solar energy into smart solar energy. In addition, we provide some directions and insights for the future development of a smart solar plant system.

The rest of the article proceeds as follows. Section 2 reflects an analysis of ML technologies for a solar plant system. In Section 2.1, we describe an ML sensor system of a solar plant based on an ML sensor model and its life cycle. Section 2.2 and Section 2.3 provide brief introductions of the main ML methods and frameworks which are being applied in solar plant systems, correspondingly. In Section 2.4, we briefly describe the open datasets and source code to create ML technologies for a solar plant system. In Section 3, we analyze, discuss, and summarize recently reported research into ML applications for solar plant systems, their advantages, and shortcomings as compared to classical methods. In addition, in Section 3, we briefly describe an ML sensor system based on a developed software that integrates with SCADA through API. The subsections of Section 3 reflect an analytical review of ML technologies for the design, forecasting, maintenance, and control of solar plants. Section 4 presents the future prospect of integrated, cutting-edge technologies, such as ML, cloud, edge computing (EC), internet of things (IoT), etc., to create a smart solar plant system that provides the digital transformation of solar energy into smart solar energy. Finally, in Section 5, we conclude the article with a brief summary of this review and a discussion about the current locus and opportunities for future development in the field of ML to create a smart solar plant system.

## 2. Machine Learning Technologies for a Solar Plant’s System

Real-life solar plant systems have complex, nonlinear dynamics due to variations in system parameters and insolation. Thus, ML methods have been proposed to approximate this complex dynamic. The recent studies [1,2,5,6,7,8,9,10,11,12,13] prove that ML technologies for a solar plant’s design, forecasting, maintenance, and control increase the effectiveness and reliability of the solar plant as compared to conventional methods. In smart sensor systems of solar plants, ML methods are crucial units to increase the quality of datasets processing the solar plant’s design, forecasting, maintenance, and control. SCADA is a control system architecture that uses sensors, programmable logic, and discrete PID controllers to control the processes of a solar plant system. The solar plant’s system includes advanced sensors. Big data from SCADA are collected 24/7. Combined with weather big data, this enables the creation of ML technologies to solve complex tasks of a solar plant’s design, forecasting, maintenance, and control.

### 2.1. ML Sensor System of a Solar Plant

Smart models based on ML technologies have the advantage of parallel computation through modern graphical processing units, which significantly decreases the time cost in SCADA datasets processing for solar plant design, forecasting, maintenance, and control [12]. 

**Figure 1:**
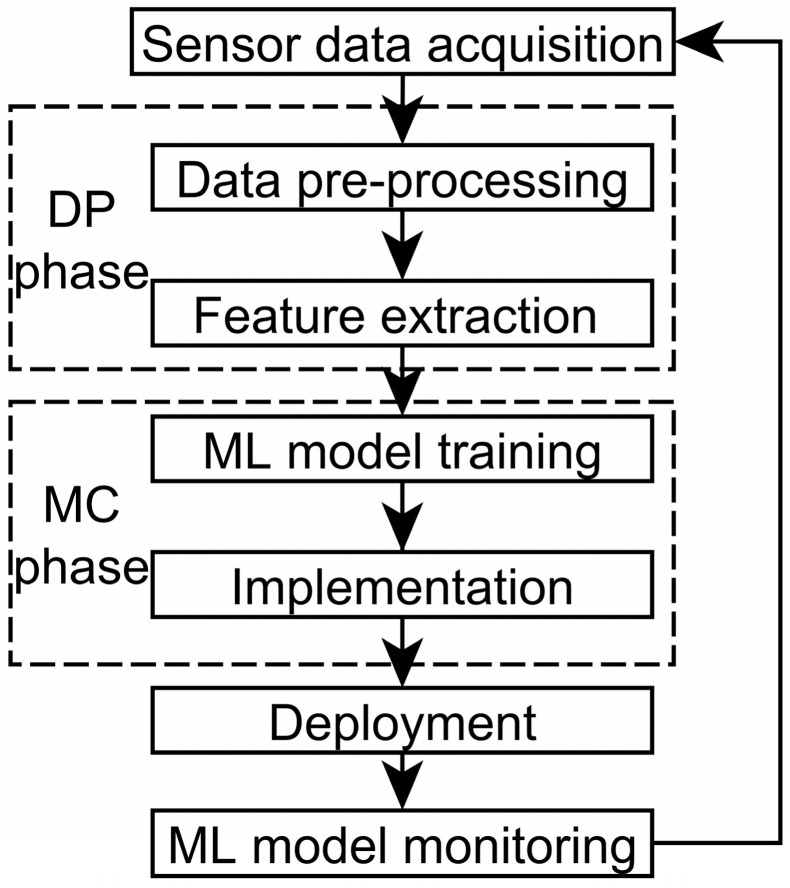
A basic life cycle of an ML sensor model.

The reliability, accuracy, and other demanded quality parameters must be composed as the performance of an ML model. This model must be created effectively with high-quality datasets to have optimal performance [14]. Figure 1 shows the basic life cycle of an ML sensor model. Smart model creation has two phases: data preparation (DP phase) and model creation (MC phase). They should be elaborated by the Cross-Industry Standard Process for Data Mining cycle (CRISP-DM) [15] and Open Neural Network Exchange (ONNX) format [16]. The CRISP-DM cycle [15] provides a pipeline for the implementation of smart models in real-time scenarios.

The sensor data of a solar plant are compiled into raw SCADA datasets. Then, these datasets are preprocessed (Figure 1) in a simple way (standardization or encoding). Data preparation methods include dimensionality reduction (principal component analysis (PCA)), sampling (subsampling, oversampling), transformation, encoding, feature extraction, and selection [14]. 

Feature extraction is a crucial step in a smart sensor system’s creation because it provides knowledge for ML model creation [14]. The DM methods generate features. The most relevant data are further separated into train, validating, and test datasets (Figure 1). An ML model to solve either classification or regression tasks is trained based on a train dataset. When a smart model provides the demanded performance, its weights are frozen. The ML frameworks, which we review in Section 2.3, provide an automatic MC phase, including validating (Figure 1). The trained ML model is deployed. If a monitored ML model does not provide optimal performance, then it is retrained based on updated datasets.

### 2.2. ML Methods for Smart Sensor Creation

An ML sensor model can be developed based on neural network (NN) or non-NN algorithms [14]. The last ones include PCA, Random Forest (RF), support vector machine (SVM), and Decision Tree (DT). In contrast with non-NN methods, NN architectures can include various neurons which are specified by ONNX [17], highly effective learning, and extracting features. A deep neural learning/network (DL/DNN), such as a recurrent neural network (RNN), convolutional neural network (CNN), and transformers, is part of the ML methods with feature learning that use multiple layers, complex connectivity architectures, and different transfer operators to automatically mine meta features from the input. NNs, such as artificial neural networks (ANNs), radial basis function neural networks (RBF-NNs), generative adversarial networks (GANs), RNNs, and CNNs have recently made major progress in practical applications of solar energy [1]. 

**Figure 2:**
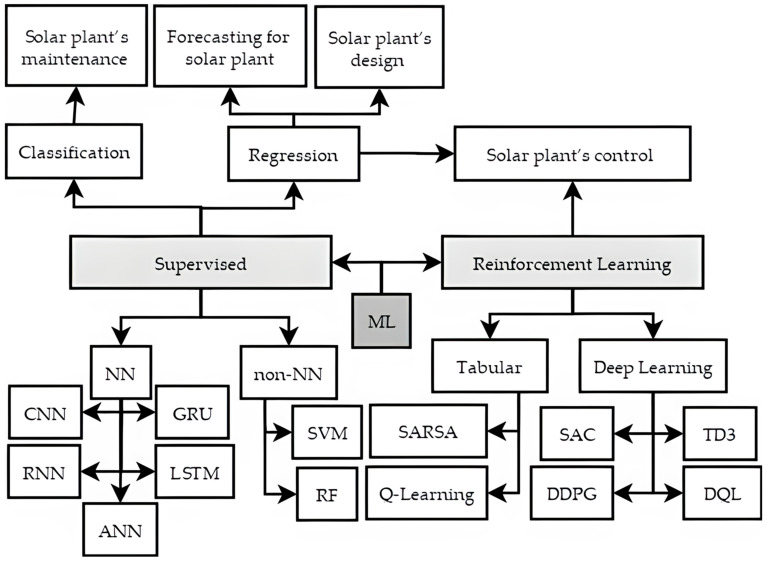
Classification of tasks that are solved based on ML methods.

Figure 2 shows two NN methods’ classes and the ML method groups according to the task they solved for a solar plant system [2].

The ensemble’s types are bagging, boosting, and stacking/blending [18,19]. Table 1 presents the comparison of ensemble techniques [18]. There are constant and dynamic weighting ensemble approaches. In recent studies, the most used ensemble methods are RF, Extreme Gradient Boosting (XGBoost), Extreme Learning Machine (ELM), etc.

Model training methods that optimize performance include quasi-Newton, stochastic gradient descent (SGD), evolutionary computation, genetic programming, etc. [15]. The creation of the ML model is the most complex and important task which includes the creation of an optimal ML model’s architecture and requires a multidimensional global optimization (GO).

The bias and variance estimate the effectiveness of a model. The improvement of a model’s bias always makes gains at the expense of variance and vice versa. The performance of ML models highly correlates with the representativeness of a dataset. A lot of techniques provide a model’s evaluation, including cross-validation, kfold, holdout with a different performance including accuracy (ACC), mean squared error (MSE), precision, receiver operating characteristics (ROC), recall, Matthew’s correlation coefficient (MCC), F1, area under the curve (AUC), mean absolute error (MAE), and root-MSE (RMSE). The relative errors, such as normalized RMSE (nRMSE), normalized MAE (nMAE), etc., facilitate the comparison between models that are tuned based on datasets with different scales.

With the goal to develop intelligent models for sizing, forecasting, and control of a solar plant system and to make an RNN more adaptive with regard to a task’s complexity and overfitting problem, we developed an MFNN [5,6,7,8]. The MFNN includes RNNs with fuzzy units and/or a convolutional block to process images. An RNN approximates a membership function in contrast to an Adaptive Network-Based Fuzzy Inference System (ANFIS). We combined the modified multidimensional quantum-behaved particle swarm optimization (PSO) with the Levenberg–Marquardt algorithm (MD QPSO) and developed a hierarchical encoder of the particle’s dimension component [5,6,7,8] to automatically create an optimal architecture of an MFNN and improve the convergence.

**Table 1:** Comparison of ensemble techniques.

Name of Method	Advantages	Disadvantages
Bagging	Tends to reduce variance more than bias	Does not work well with relatively simple models
Boosting	Reduces bias and variance	Sensory to noise and outliers in data. Susceptible of overfitting
Stacking/blending	Provides the optimal combination of base learners, reduces variance, and bias [18]	In the case of huge datasets, the computational time increases sufficiently as each classifier is working independently on the huge dataset.

We implemented an MFNN and its life cycle, which includes automatic creation and self-adaptation as an intelligent framework based on the authors’ software [20]. This intelligent framework provides the automatic creation of the optimum architecture of an MFNN with regard to a task’s complexity. 

All the above-mentioned ML methods and algorithms were implemented as software by an ML Framework, which represents a tool to create a smart sensor system.

### 2.3. ML Frameworks

ML frameworks implement many ML methods [15]. Table 2 shows the comparison of popular ML frameworks.

Big data ecosystems, namely Apache Flink, Apache Spark, and Cloudera Oryx 2, include built-in ML libraries for large-scale DM. These ML libraries evolve presently, but the potency of the entire ecosystem is significant.

Google, Facebook, and Microsoft developed most of the DL frameworks that support ONNX, namely PyTorch, TensorFlow, Caffe2, Microsoft CNTK, and MXNet.

Chainer, Theano, Deeplearning4, and H2O are also appropriate DL libraries and frameworks for smart sensor system creation.

The high-level DL wrapper libraries such as Keras, TensorLayer, and Gluon are developed on top of the DL frameworks. They provide a simpler but more computationally expensive way for smart sensor system creation.

The ML frameworks provide an automatic MC phase of an ML model, including validating (Figure 1). An ML sensor system can be implemented as software based on an ML framework that supports ONNX. Such implementation will provide flexibility and all an ML framework’s advantages for a developed ML sensor system.

**Table 2:** Comparison of ML frameworks.

Name	Advantages	Disadvantages
TensorFlow	Open-source, API-oriented, cross-platform, ML/DM toolbox implements many ML methods.	The code is not flexible. Lack of documentation. Toolbox oriented for academic usage.
Microsoft CNTK	Open-source, fast-evolving, supports ONNX, supported by Microsoft.	Limited facilities for mobile platforms.
Caffe2	Cross-platform, supports mobile platforms, supports ONNX.	Complex as compared to PyTorch. Without dynamic graph computation.
PyTorch	Dynamic computational graph. Automatic implementation of ML models. Supports ONNX.	Absence of monitoring and visualization tools like a tensor board
Keras	Open-source, provides backend tools from Google and Microsoft. Detailed specification. API for DL. Quick implementation of DL models (e.g., TensorFlow, Theano, CNTK).	Modularity and simplicity make gains at the expense of flexibility. Limited facilities to create a new architecture.

### 2.4. Open Resources for ML Research in a Solar Plant System

The open solar energy data sources, including big data, provide the development of cutting-edge ML technologies in solar energy. 

The GitHub repositories [21,22] are implementations of maximum power point tracking (MPPT) systems [21] and management of cities’ demand/load [22] based on an open-source Gym toolkit [23]. An open-source tool pymgrid [24] provides the creation and simulation of various microgrids. Octave [25] and Scilab [26] are open sources that are compatible with MATLAB. 

Table 3 presents a brief description of the open datasets to implement and validate ML solar plant systems. 

## 3. Machine Learning Applications for a Solar Plant System

**Figure 3:**
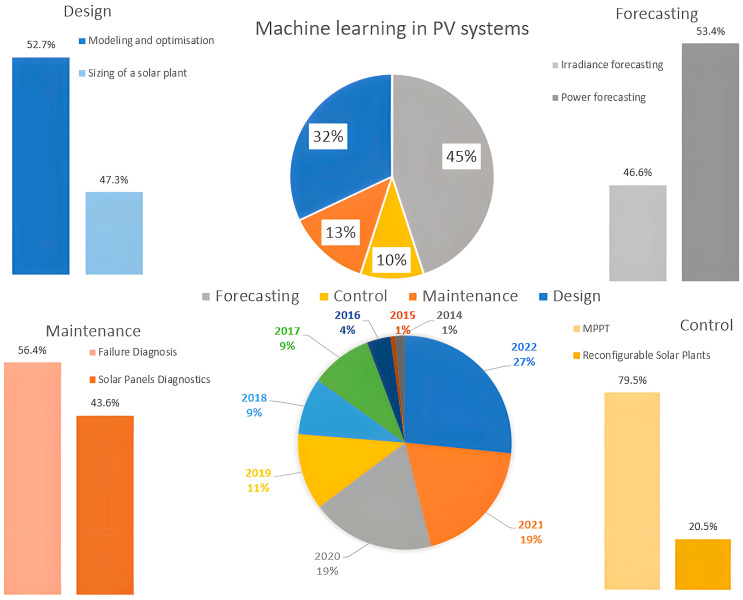
ML technologies for a solar plant’s design, forecasting, maintenance, and control.

This section presents a review of research studies that have been published mostly in the last five years on the topic of ML applications for a solar plant. The literature review process elaborates on the articles’ search queries in Scopus/ScienceDirect, IEEEXplore, ResearchGate, and Google Scholar with the following keywords: machine learning, neural networks, DL, PV, and solar plant. We focused on four important tasks’ categories in the solar plant systems, as shown in Figure 3: design, forecasting, maintenance, and control. We are persuaded that tasks of these categories are most in demand in solar plant systems where ML can be applied with high efficiency. Figure 3 identifies the number of publications devoted to ML for a solar plant’s design, forecasting, maintenance, and control that have been published mostly during the last five years. We prepared the data based on the considerable contributions from the most cited journals. We have not covered cybersecurity in a solar plant system since it was covered in-depth in study [1].

**Figure 4:**
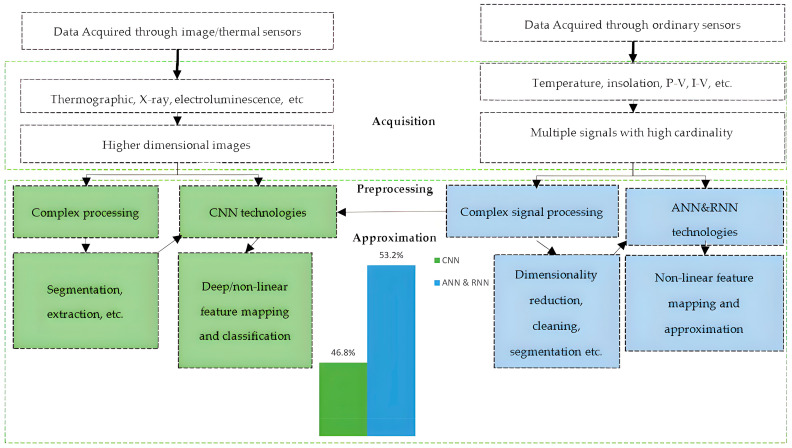
Classification of ML sensor types for a solar plant system.

Figure 4 reflects the number of publications devoted to CNN, ANN, and RNN technologies for a solar plant system that have been published mostly in the last five years. Figure 4 also presents the various types of feature spaces- to create a smart sensor system based on an ML method. It specifies the essential preprocessing and ML models to create a smart sensor [14]. 

**Figure 5:**
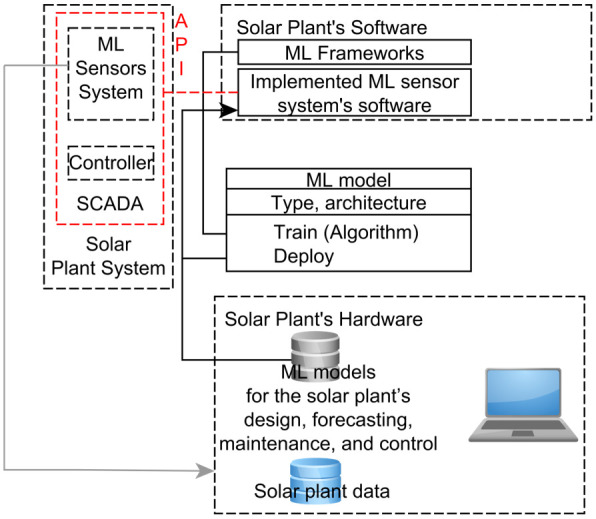
Solar plant system based on ML technologies.

The researchers in [10] noted that a pipeline implementation of an ML system is demanded. Therefore, we proposed a simple but effective pipeline scheme of an implementation (implementation step in Figure 1) of an ML sensor system for a solar plant. Figure 5 shows this simple scheme of a solar plant system based on ML technologies for a solar plant’s design, forecasting, maintenance, and control. The center of a solar plant controller controls all devices and data of the solar plant and congregates datasets from the sensors, meteorological stations, and inverters [3]. The SCADA system is able to integrate sensor systems and ML technologies into an ML sensor system based on software that implements ML sensor models and integrates with SCADA through API. This software through API can transmit a control signal which is generated by an ML sensor model to a solar plant controller [14]. These ML sensor models for a solar plant’s design, forecasting, maintenance, and control are implementations of a basic ML model class which is represented in Figure 5 as a UML class diagram. A method “Train” of a basic ML model class implements the MC phase, including validating. 

Thus, the impact of the ML technologies based on the proposed scheme (Figure 5) on a solar plant value chain will mostly be associated with the cost of software development (including API development and the developed software’s integration with SCADA) and maintenance. This developed software implements an ML sensor system based on an ML framework that supports ONNX. Most ML systems, which we review in subsections of Section 3, can be implemented on a solar plant based on the proposed scheme. Such implementation will provide flexibility and all ML framework’s advantages for the developed ML sensor system and its digital transformation into a smart sensor system which we outlined in Section 4.

### 3.1. ML Technologies for Design of the Solar Plants

The optimal design of a solar plant is a very complex task that requires the fulfillment of models for a solar plant’s components as well as the usage of global optimizers.

#### 3.1.1. Parameter Identification in a Solar Plant System

The parameter extraction models for the single (SDM), double (DDM), or triple diode solar cell model (TDM) with RMSE as the performance metric are highly demanded for simulation and fault detection of a solar plant system. 

In studies [35,36], the ML parameter identification models for SDM provided good performance. There are many heuristic search algorithms, including bioinspired, that were adapted to solve the parameter identification task of the different solar cell models [37,38,39,40,41,42,43,44,45,46,47,48,49,50]. Table 4 displays a brief comparison of the parameter identification models from studies [35,36,37,38,39,40,41,42,43,44].

In [45], the parameter identification models for 17 different industrial solar cells/modules are reported. The hybrid bee pollinator flower pollination algorithm (BPFPA) [46] has the lowest RMSE and highest convergence as compared to all 21 reviewed parameter identification metaheuristic algorithms. Table 5 summarizes the comparative results of papers [42,43,45,46,47] to set benchmarks for the performance comparison of the parameter identification models based on different metaheuristic algorithms for the 57 mm dia RTC France solar cell.

Summarizing, we highlight a need to assess more benchmarks for a performance comparison of the parameter identification models including ML methods. 

#### 3.1.2. Sizing of a Solar Plant

Within the research literature, a whole array of differing sizing methods for a solar plant has been proposed. These sizing methods of a solar plant are classified as intuitive, numerical, and analytical algorithms. The intuitive algorithms do not provide effectiveness and reliability. The numerical algorithms require a long time series of insolation. Many of the analytical algorithms use a concept of the system’s reliability or loss of load probability. ML technologies provide an estimation of the optimal number of panels, storage capacity of batteries, tilt, and azimuth angles for a solar plant. Moreover, several ML technologies have been developed to size a solar plant. Table 6 shows a brief comparison of ML sizing methods of a solar plant [5,51,52,53,54,55].

Summarizing, we highlight a need to assess more benchmarks for a performance comparison of the PV sizing ML models. In addition, DL methods, including RNN, that extract knowledge from time series and effectively approximate insolation and load under small disturbances of a PV system dynamic, including degradation, are promising alternatives.

### 3.2. ML Technologies for Insolation and Power Forecasting of Solar Plants

Energy production of a solar plant is highly dependent on weather conditions such as insolation and temperature. Thus, it is difficult to balance the production and consumption of the electric grid with integrated solar plants where production levels fluctuate. In case of a deviation from an hourly plan schedule of solar plant power, the energy market charges penalties. Hence, many ML methods have been implemented to forecast insolation and the output power from a solar plant. 

**Figure 6:**
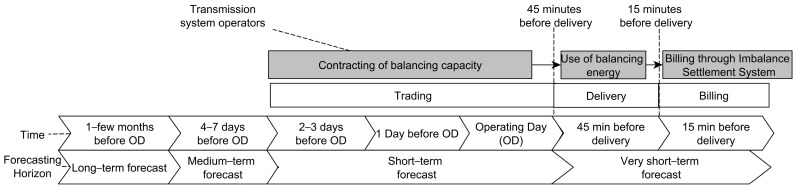
Specifics of the energy market to forecasting and classification of ML forecasting models.

Figure 6 presents specifics of the energy market to forecasting and classification of ML forecasting models based on a forecasting horizon [1,56].

The surveys of insolation and power forecasting of a solar plant in [57,58,59,60,61,62,63] appraise various approaches and methods to increase the performance of forecasting models under uncertainties. According to the reviews, ANNs are the most popular method for forecasting, as they are easy to implement and quite effective as compared to classical methods, such as conventional autoregressive integrated moving average (ARIMA), etc.

#### 3.2.1. ML Technologies for Power Forecasting of Solar Plants

The power forecasting of a solar plant provides safety and effectiveness of grid control. There are mainly three ways to power forecast for a solar plant:

- only historical output power recorded is used,
- forecasted meteorological parameters are used as input,
- combination of the historical power data with forecasted meteorological parameters is used.

Recent studies present the ML methods which effectively forecast a solar plant’s power.

The study [64] reveals that the output power with the insolation and the air temperature has a linear and nonlinear correlation, correspondingly. Recently, researchers have been more interested in the ML application to increase the accuracy of the forecasters [61,65,66,67,68,69,70,71,72,73,74,75,76,77]. 

The simple (in [61], preprocessing generated normalized insolation; in [73], preprocessing elaborated k-means) and complex data preprocessing algorithms (in [71], four CNNs with different filters mine simple features from a sequence of time series; a single-kernel CNN mines the meta features from the simple features) provide for the ML model better performance (Table 7).

Due to forecast power, in [69,70], researchers integrated a PV-performance model into ML methods such as RF, SVR, CNN, LSTM, and hybrid CNN-LSTM. The results indicated that the proposed ML models provide the best performance regardless of the model’s type and forecasting horizon (Table 7).

Table 7 shows that indirect, very short-term forecasting ML models [61,67] provide higher accuracy as compared to direct ones.

Table 7 shows that the dataset’s length has a positive correlation with forecast performance (an average correlation coefficient of normalized corresponding columns is 0.34). Table 7 displays that the forecast horizon has a negative correlation with forecast performance (an average correlation coefficient of normalized corresponding columns is −0.31).

**Table 7:** The performances of the power forecasting ML models.

Predicting Method	The Forecasting Horizon	Dataset’s Length	RMSE (Wh/m^2^)	RMSE%
Stack-ETR (TF) [77]	1 day	4 years	37.37	-
Stack-ETR (MC) [77]	1 day	4 years	13.95	-
Stack-ETR (PC) [77]	1 day	4 years	20.41	-
Stack-GBDT [78]	1 day	4 years	47.7826	-
RNN-LSTM (TF) [79]	1 day	4 years	39.2	-
RNN-LSTM (MC) [79]	1 day	4 years	19.78	-
RNN-LSTM (PC) [79]	1 day	4 years	26.85	-
XGBoost-DNN [80]	1 day	10 years	51.35	-
DPNN [81]	1 day	2 weeks	52.8	-
K-means-AE-CNNLSTM [82]	1 day	-	45.11	-
LSTM-RNN [83]	1 day	1 year	82.15	-
LSTM [84]	1 day	-	139.3	-
ELM (TF) [85]	1 day	1 year	90.41	-
ELM (MC) [85]	1 day	1 year	59.93	-
ELM (PC) [85]	1 day	1 year	54.96	-
ANN’s ensemble [60]	1 h	-	5	6.25%
MLPNN [62]	1 day	1 year	160.3	-
TDNN + clustering [62]	1 day	1 year	122	-
MLFFNN based on BP [62]	1 day	1 year	223	-
CNN-Simple [65]	1 day	6 years	51	-
Multi-headed CNN [65]	1 day	6 years	81	-
CNN-LSTM [65]	1 day	6 years	51	-
5D CNN-LSTM [67]	10 min	1 year	0.083	-
	30 min	1 year	0.22	-
	60 min	1 year	0.45	
	90 min	1 year	0.72	
	120 min	1 year	1.05	
	150 min	1 year	1.44	
	180 min	1 year	2.05	
D-PNN [68]	1 day		60	-
RF [69]	1 h	15 months	-	11.83%
Support Vector Regression (SVR) [69]	1 h	15 months	-	13.71%
CNN [69]	1 h	15 months	-	15.27%
LSTM [69]	1 h	15 months	-	14.89%
Hybrid [69]	1 h	15 months	-	15.72%
RF [70]	24 h	15 months	-	7.58%
	48 h	15 months	-	7.75%
	72 h	15 months	-	7.93%
SVR [70]	24 h	15 months	-	8.06%
	48 h	15 months	-	8.21%
	72 h	15 months	-	8.29%
CNN [70]	24 h	15 months	-	8.69%
	48 h	15 months	-	8.86%
	72 h	15 months	-	9.16%
LSTM [70]	24 h	15 months	-	7.56%
	48 h	15 months	-	8.08%
	72 h	15 months	-	8.12%
Hybrid [70]	24 h	15 months	-	8.06%
	48 h	15 months	-	8.69%
	72 h	15 months	-	8.96%
Quad-kernel deep CNN (QKCNN) [71]	10 min	-	-	4%
SVR-RBF [72]	1 h	-	10	-
Deep RNN [72]	1 h	-	5	-
BackPropagation NN [73]	1 day	100 days	3.66	-
LSTM NN [74]	1 day	3 months	7.1	-
RNN [74]	1 day	3 months	9.2	-
Generalized regression neural network (GRNN) [74]	1 day	3 months	13.1	-
Extreme learning machine (ELM) [74]	1 day	3 months	24.1	-
Transfer learning constrained LSTM (TL + C-LSTM) [76]	1 day	1 year	8.89	-
MFNN [5]	2 day	3 years	43.15	20.15%
RFR [77]	1 day	4 years	38.96	-
XGB [77]	1 day	4 years	34.11	-
DTR [77]	1 day	4 years	36.61	-
ADA [77]	1 day	4 years	35.52	-
ETR [77]	1 day	4 years	32.05	-
Stack-RFR [77]	1 day	4 years	24.9	-
Stack-ETR [77]	1 day	4 years	23.09	-
Stack-ADA [77]	1 day	4 years	24.58	-
Stack-XGB [77]	1 day	4 years	23.97	-

#### 3.2.2. ML Technologies for Insolation Forecasting of the Solar Plants

ML technologies for insolation forecasting provide great benefits to smart grid integration and solar plant management. ML insolation forecasting is a necessary step for indirect power forecasting that provides higher accuracy as compared to a direct one. Thus, output of an insolation forecasting ML model can be used as an additional input signal for an indirect power forecasting ML model. 

In Table 8, we briefly summarize the insolation forecasting ML models from studies [5,7,60,62,65,67,68,69,72,77,78,79,80,81,82,83,84,85].

Table 8 shows that the dataset’s length has a positive correlation with forecast performance (an average correlation coefficient of normalized corresponding columns is 0.34). Table 8 displays that the forecast horizon has a negative correlation with forecast performance (an average correlation coefficient of normalized corresponding columns is −0.31).

Summarizing, we highlight a need to assess more datasets and benchmarks for the performance comparison of ML technologies for insolation and solar plant power forecasting. The number of data preprocessing algorithms has a negative correlation with a forecast’s performance. The dataset’s length and forecast horizon have positive and negative correlation with a forecast’s performance, correspondingly. A one-year test dataset is enough to create and validate a robust ML model. Indirect power forecasting provides higher accuracy as compared to a direct one. In addition, DL methods including transformers based on an attention mechanism that hierarchically preprocess and mine knowledge from datasets are promising alternatives.

**Table 8:** The performances of the insolation forecasting ML models.

Site	Model	MBE [W/m^2^]	RMSE [W/m^2^]	R^2^	Dataset’s Length	Horizon
Caruru	RF [31]	0.9309	9.1715	0.9962	11 years	30 min
	ANN [31]	3.1310	7.006	0.9977	11 years	30 min
Barrancominas	RF [31]	0.0568	9.1002	0.9961	11 years	30 min
	ANN [31]	3.0637	6.9222	0.9977	11 years	30 min
Chajal	RF [31]	0.3947	7.0558	0.9976	11 years	30 min
	ANN [31]	2.6189	6.2072	0.9981	11 years	30 min
Sipi	RF [31]	0.6185	7.8242	0.9972	11 years	30 min
	ANN [31]	2.7263	6.3490	0.9982	11 years	30 min
Puerto Merizalde	RF [31]	0.5521	7.9230	0.9971	11 years	30 min
	ANN [31]	2.8704	6.6222	0.9979	11 years	30 min
Bogota	RF [31]	0.6464	7.7266	0.9973	11 years	30 min
	ANN [31]	2.6964	6.3453	0.9981	11 years	30 min
Narino state	LSTM [86]	-	42	-	11 years	1 day
	LSTM [86]	-	64	-	11 years	1 week
Tetouan, Morocco	SVM [87]	34.709	13.59	-	3 years	1 day
	ANN [87]	23.883	15.8		3 years	1 day
Bangladesh	RNN [88]	-	0.958	-	6 years	1 h
	LSTM [88]	-	1.14	-	6 years	1 h
	GRU [88]	-	0.891	-	6 years	1 h
Abakan, RF	MFNN [5]		21.5	0.91	2 years	2 day
Ghardaia, Algeria	LSTM [89]	-		0.98–0.96	3 years	1–12 h
Uluru (Ayers Rock) in Australia	ShuffleNet [90]	-	0.1471	-	2 years	1 h
	SqueezeNet [90]	-	0.1146	-	2 years	1 h
	ResNet-18 [90]	-	0.0941	-	2 years	1 h
	GoogLeNet [90]	-	0.0850	-	2 years	1 h
	AlexNet [90]	-	0.0729	-	2 years	1 h
	CEEMDAN-AG-RE-EML [90]	-	0.0642	-	2 years	1 h

### 3.3. ML Technologies for Maintenance of Solar Plants

ML methods solve the most complex tasks, which include failure classification, detection, localization, and automated solar panel diagnostics, based on solar plant sensor data (Figure 4). Thus, grid operators can greatly increase the effectiveness and reliability of their solar plants based on ML technologies. 

ANN, FL, DT, RNN, RF, and different ensembles automatically detected basic solar plant faults based on data from ordinary sensors (Figure 4). DL and various types of CNN automatically perform analysis of infrared (IFR) images that are tracked by Unmanned Aerial Vehicles (UAVs). In this field of research, usually a dataset is highly unbalanced, i.e., it has unlabeled data and/or has rare failures. For this reason, the Balanced Accuracy, F1 score, Cohen’s Kappa, or MCC better reflect the model’s performance as compared to traditional accuracy metric.

Most of the ML models were created based on the dataset which was generated from simulation. A limited number of failure classes were considered, with the exception of a number of works in [91,92] in which 10 or more faults were considered (Table 9).

#### 3.3.1. ML Technologies for Failure Diagnosis of the Solar Plants

According to study [93], there are six different categories of solar plant systems failures: shading, open-circuit, degradation, line-to-line, bypass diode, and bridging. 

Frequent faults are failure in a component, system isolation, inverter shutdown, shading, and inverter MPP. In recent years, ML techniques that process data from ordinary sensors (Figure 4) have been highly applied for fault classification and, in some cases, to identify the location of a failure. 

In studies [91,92,94,95,96,97,98], researchers detect, classify, and localize [98] different failures of a solar plant system based on non-NN [91,92,95,97], ANN [97], ANFIS [98], and LSTM [94] that simply process signals from ordinary sensors (Figure 4(1)). 

In studies [99,100,101,102,103,104,105], researchers detect, classify, and localize [100] different failures of a solar plant system based on CNNs. For this purpose, researchers tuned CNNs based on the created dataset which sample represented a two-dimensional or three-dimensional transformation of data from ordinary sensors (Figure 4) namely, a scalogram [101], a two-dimensional time series graph [99], a three-dimensional image [103] and a polar-coordinate image [105]. This transformation can be simple (in [99], only PV current and voltage were composed into a two-dimensional time series graph) or complex (in [103], the direct current and alternating current values of a PV system were composed into a three-dimensional image based on a Gramian Angular Field; in [105], the time domain waveform signals were composed into a polar-coordinate image based on a symmetrized dot pattern (SDP)).

We proposed a failure forecasting system of a wiring losses’ failure free operating period of a PV box based on an MFNN that has two RNNs with fuzzy units [5]. We created the MFNN based on a two-year historical dataset which included 20 kW PV array’s signals. The developed fault forecasting system of the solar plant based on the tuned MFNN effectively forecasted a wiring losses’ failure free operating period of a PV box. The relative error of the tuned MFNN was 0.0006.

In Table 9, we summarize the ML models for PV failure diagnosis from studies [91,92,94,95,96,97,98,99,100,101,102,103,104,105].

**Table 9:** Summary of the ML models for PV failure diagnosis.

Fault Diagnosis Stage			Types of Faults	Performance (%)	Specific Data/Method (s) Applied/Ref.
Det	Clas	Loc			
✓	✓	-	Inverter fault, grid anomaly, mismatch fault, MPPT fault, converter fault	False alarms < 1. Computational time is 11.809 s	PCA-KDE-based multivariate KL divergence/[91]
✓	✓	-	Degradation, PS, PS w/BpD, short circuit, open, PS w/BpD short	98.3	Experimental data/ stacked autoencoder/[92]
✓	✓	-	line-to-line	97.66	Data with noise/
			hot spot	98.78	LSTM/[94]
✓	✓	-	line-to-line, open circuit, degradation, and PS	99 accuracy that is superior as compared to DT	Dataset that was created during simulation/ RF/[95]
✓	✓	-	PS, bridging, bypass diode, temperature, short circuit, and complete shading	99.91 performance, which is superior as compared to DTs, XGBoost and RF	Dataset with 1200 samples /ANNs /[96]
✓	✓	-	Healthy mode	98.17	Dataset with 586,580 samples/PCA + RF/ [97]
			inverter fault	99.93	
			grid connection fault	99.93	
			sensor fault	99.96	
			panel fault	100.0	
			panel connection fault	100.0	
✓	✓	-	PS, open circuit, line-to-line, arc	70.45	Scalograms with noise/CNN/[101]
			Open-circuit, line-to-line,	Average accuracy 99	2-D time series graph/CNN/[100]
✓	✓	✓	PS w/ BpD, PS w/ reversed BpD, short circuit, increase series resistance	99.94 for Classification, 99.54 for Location	CNN w/residual GRU/ [100]
✓	✓	✓	Line-to-line, open-circuit, short-circuit	R = 0.9989, RMSE = 0.0383	ANFIS Sugeno/[98]
✓	✓	-	Short circuit, PS, abnormal aging, and hybrid failures [103]	98.41	CNN and a fully connected module/[102]
✓	✓	-	PS, degradation of a TF module, short circuit, open circuit	Average accuracy 95.78 which is superior as compared to CNN	Test dataset/ResNet/ [103]
✓	✓	-	Line-to-line	100.0	Dataset of 3D images/ 3D CNN/[104]
			shorted modules in strings	91.67	
			open module in strings	91.67	
			shorted strings in arrays	100.0	
			open strings in arrays	95.24	
			healthy mode	100.0	
✓	✓	✓	Normal PV module	100.0	Dataset includes 3200 samples that generated by SDP, test dataset includes 800 samples (200 samples of each failure)/CNN/[105]
			poor connection on a PV Module	100.0	
			PV module breakage	100.0	
			bypass diode	99.5	

Summarizing, we highlight a need for open datasets to assess experimental results on real testbeds and an open tool to generate and process scalograms based on transformers with an attention mechanism which feasibly outperforms other ML methods, such as CNNs. For failure detection and classification, there is a need to study the MPPT algorithms based on Reinforcement Learning (RL) and a spiking neural network under failure conditions.

#### 3.3.2. ML Technologies for Solar Panel Diagnostics

The drop in solar plant productivity due to deviant maintenance modes caused by nonclean module surfaces, cell damage, delamination, or hot spots, demands a solar panel diagnostic based on the ML image sensors that process the panels’ images (Figure 4).

In studies [106,107,108,109], researchers localized and identified different failures of a solar plant system based on CNNs that process the solar panels’ images, including thermographic images [106,107,108]. In Table 10, we summarize the ML technologies for PV diagnostics from studies [106,107,108,109,110,111,112].

Summarizing, we identify an opportunity to collect and make datasets available in which new ML models for solar plant diagnostics can be tested. In the reviewed studies, a considerable number of smart sensors process images almost perfectly. In the reviewed studies, the smart sensors where signals of image sensor and the CNN blocks strongly correlate provide high performance. There is an argumentative direction to substitute non-NN smart models with a DNN-based model for the solar plant’s maintenance because DNN provides better information processing quality and performance as compared to non-NN smart models. In addition, ML methods such as GANs can be applied to generate artificial thermal images and create knowledge of the failure. Moreover, future research can comprise the elaboration of a pipeline for implementing a real time solar plant diagnostic system based on DNN or spiking neural network. 

### 3.4. ML Technologies for Control of Solar Plants

The application of ML methods for the MPPT of solar plant systems has massive potential to increase their stability, reliability, dynamic response, and other essential advancements and easing their integration to electric grids.

#### 3.4.1. ML MPPT Technologies of Solar Plants

The insolation and cell temperature of solar panels primarily define the total generated power by a solar plant. In the research reviews, a whole array of differing MPPT algorithms has been revealed [1,2,4]. Among them, the perturbation and observation (P and O) and incremental conductance (INC) algorithms are the most popular due to their easy and simple implementation. However, controllers which were created on the basis of these algorithms for solar plant systems have very bad speed of the response times, a long time to settle down from oscillating around the reference state. Furthermore, under PS, the MPPT task demands GO. Thus, traditional methods for MPPT do not provide global MPPT (GMPPT) and decrease efficiency in solar power production. 

There are a lot of GO algorithms to create a GMPPT model [1,2,4], but all these models have the following disadvantages: power oscillations in the calm mode; the initialization is a critical issue that decrease power; very slow convergence to a GMPP under insolation’s variation, etc. Due to all the above-mentioned disadvantages, GO-based, real-time GMPPT of a solar plant are ineffective while ML technologies provide the required performance. 

In Table 11, we summarize the ML models for MPPT of a solar plant from studies [8,113,114,115,116,117,118,119,120,121,122,123,124,125,126].

In [114], researchers integrated the trained RL control agent into a fuzzy-logic-sliding mode control and incremental conductance-sliding mode control (RL FL INC) and gained better performance as compared to a classical RL agent (Table 11).

In [120], researchers created an MPPT controller based on a fuzzy logic search of variable voltage step size and fuzzy adaptive RBF-NN. The simulation results reflect the superiority of the developed MPPT controller as compared to the conventional P and O and RBF-NN.

**Figure 7:**
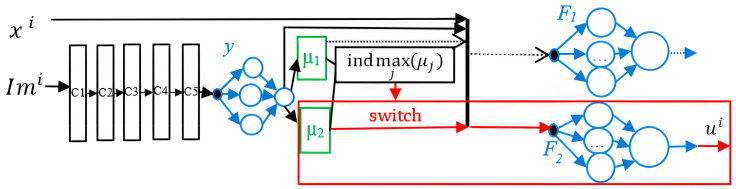
The GMPPT system based on an MFNN.

In [121], we introduced the GMPPT system based on an MFNN that has five convolutional blocks to process the PV array’s images, RNNs, and fuzzy units. Figure 7 shows the proposed GMPPT system based on an MFNN, where $Im^{i}$ is image of solar plant’s modules; $x^{i}=\left(\;V^{i},\;P^{i-1},\;dI/\;dV^{i}\right)$ and $u^{i}$– input and output signal of MFNN, correspondingly; $\mu_{j}$ —membership function of the fuzzy sets $A_{j}$ ($A_{1}$ is the rapidly increased uniform insolation, $A_{2}$ is nonuniform insolation); $z=\mathrm{ind}\underset{j}{\max}(\mu_{j})=\left\{j\;\right|\forall k\neq j\;\mu_{j}\geq \mu_{k}\}$ triggers the rule, which corresponds the z fuzzy set and RNN $F_{z}$. The performance and control speed in GMPPT under PS of the created MFNN were superior as compared to the PSO and RNNs.

**Table 11:** Recent comparative studies of ML-based and other MPPT implementations.

ML Method for MPPT	Software Platform	MPPT Simulation Time (s)	Steady-State Oscillation (%)	MPPT Efficiency (%)
RL control agent [113]	Simulink	-	Almost zero	99.4
RL FL INC [114]	MATLAB/Simulink	1	-	99.8
Q-learning [115]	MATLAB and Simulink R2015a	20	-	-
Q-learning [116]	MATLAB/Simulink	30		98.97
Q-learning (DQL) agent [117]	MATLAB/Simulink	8	±2	97
Deep deterministic policy gradient MPPT [118]	MATLAB and Simulink	40	-	97.5
Q-table MPPT [119]	MATLAB and Simulink R2017b	40	-	97.5
Fuzzy Adaptive RBF-NN [120]	MATLAB/Simulink	1.5	Almost zero	99.21
MFNN [121]	Authors’ software [20]	8	Almost zero	99.3
DL RL agent [122]	OpenAI Gym environment [21,23]	10	-	99
Bayesian ML (BML) [123]	MATLAB 2013a/Simulink	30	Almost zero	98.9
ANN [124]	MATLAB/Simulink	10	-	99
Feedback Linearization (FBL) embedded Full Recurrent Adaptive NeuroFuzzy (FRANF) [125]	MATLAB/Simulink	25	-	90.2
Hermite Wavelet-embedded Neural Fuzzy [126]	MATLAB/Simulink	12		94.04

Summarizing, we highlight a demand for implementing more benchmarks for performance comparison of the real-time MPPT ML models based on ML frameworks, which we presented in Section 2.3. In addition, a real-time MPPT model based on a spiking neural network is a promising alternative.

#### 3.4.2. ML Technologies for Control of Reconfigurable Solar Plants

The technology of reconfigurable PV arrays (rPV) by switching the electrical interconnection maximizes the generated PV array power in case of PS [127,128]. There are two classes of rPV: static and dynamic. Researchers proposed a lot of rPV’s structures, including Honey Comb, Series Parallel, Total Cross Tied (TCT), etc. [127,128,129,130,131,132,133,134,135,136,137,138,139]. According to the articles [140,141,142], the last one generates more power in case of PS as compared to other structures. The GMMPT of an rPV array in case of PS represents a GO task.

In Table 12, we summarize the ML models for rPV from studies [130,131,132,133,134,135,136].

The comparative analysis of recent rPV methods in [130] revealed that a TCT rPV based on a Static Shade Dispersion Physical Array Relocation (SD-PAR) algorithm and Modified Harris Hawks Optimizer (MHHO) algorithm that generated a switching matrix generates more power under PS as compared to other methods. Although, all metaheuristic optimizers do not provide a GMMP in real time mode because of a slow convergence.

**Figure 8:**
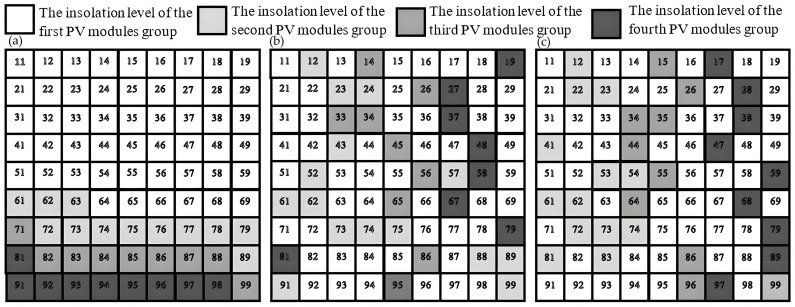
The configuration scheme created by (**a**) the TCT rPV, (**b**) the rPV system based on the MFNN, and (**c**) the rPV system based on GA.

**Figure 9:**
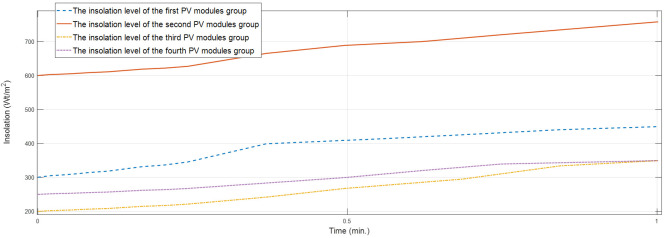
The solar panels groups’ insolation.

**Figure 10:**
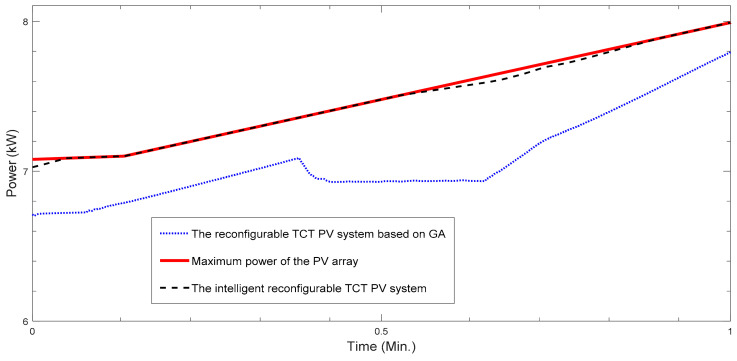
Curves of the generated power of rPV system based on the MFNN and GA.

The goal of study [136] is a GMPPT of an rPV array based on the MFNN in a case of PS. We created an optimal MFNN based on the dataset that contains the 20 kW PV array’s signals under PS including PV array images that were congregated at the town Abakan from 31 January 2018 through 31 December 2018. Figure 8 and Figure 9 display the insolation of the four solar panels’ groups for the time period 9:20 am 3 December 2018–9:21 am 3 December 2018. Figure 10 shows that the rPV system based on the MFNN outperforms an rPV system based on GA because last one does not provide GMPP in this case. Similarly, we evaluated the performances of the rPV system based on the MFNN and rPV system based on GA on 100 test samples from the time period 1 December 2018–31 December 2018. The comparative simulation results show the superiority in terms of robustness and control speed of the created intelligent rPV system under PS that provides on average 30% more energy as compared to a TCT rPV system based on GA.

**Table 12:** Comparison of ML Technologies for Control of the Reconfigurable Solar Plants.

ML Technology	Advantages/Disadvantages	Performance
TCT rPV based on Static Shade Dispersion Physical Array Relocation (SD-PAR) algorithm and Modified Harris Hawks Optimizer (MHHO) [130]	Disadvantage: GO-based, real-time GMPPT of a solar plant are ineffective because of the slow convergence	Technology generates more power under PS as compared to other methods.
Reconfiguration methods based on a GA [129,131]	Disadvantage: GO-based, real-time GMPPT of a solar plant are ineffective because of the slow convergence	The simulation results in Simulink for TCT rPV revealed that the developed method increased power: by 16.68% and 6.8% in three PS scenarios as compared to the TCT and the Su Do Ku scheme [129]; in four PS scenarios as compared to TCT.
ANFIS and an OCS [132,133]	-	Created method provided faster GMMPT and an average of 21% more generated power as compared to the P and O algorithm
Fuzzy controller [134]	Disadvantage: the proposed scheme does not provide MPPT under dynamic PS due to constant threshold-based switching of a fuzzy controller.	-
CNNs [135]	Advantage: Eight CNNs are implemented by PyTorch and validated on 1842 images under four PS scenarios	The VGG 19 provides the best result (MAPE is 3.75%, RMSE is 0.0513, accuracy is 88.47%).
MFNN that contains: a convolutional block, RNNs and fuzzy units [136]	Advantage: MFNN is implemented by authors software [20]. The trained MFNN by processing of the signals from ordinary sensors and PV array’s image creates the GMMP interconnection matrix and GMMP voltage in case of PS.	The results show the superiority of the created intelligent rPV system under PS in terms of robustness, control speed that provides on average 30% more energy, as compared to a TCT rPV system based on GA

Summarizing, we identify an opportunity to use RNN for rPV that provides a GMMP interconnection matrix and GMMP voltage under dynamic PS. Nevertheless, an rPV’s payback period is about 20 years [127] solely in places where PS happens daily, or over the full year leastwise in the seasons where solar production is great.

## 4. Future Technologies for Smart Solar Energy

**Figure 11:**
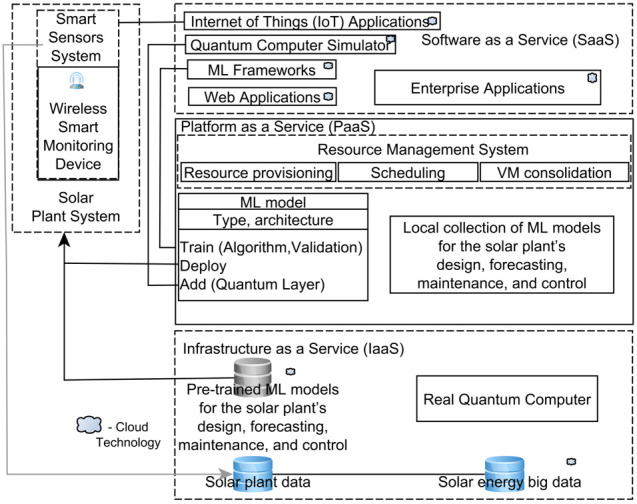
Smart solar plant system.

The long-term contribution, including increased capacity of solar energy, depends on solving the remaining tasks of grids integration, high costs, and low efficiency, mainly through the research and development of a smart solar plant system based on integration of cutting-edge technologies, including DNN [137,138,139,140,141,142,143,144,145,146,147]. To attain the smart optimization and high efficiency of solar energy, the cloud, big data, ML, EC, IoT, quantum, and sensor technologies need to be adaptively combined and implemented as smart grid, home, and city applications. Figure 11 reflects the overlapping integration of these technologies into a smart solar plant system. The integration of the above-mentioned cutting-edge technologies provides high efficiency of ML technologies for the solar plant’s design, forecasting, maintenance, and control. Implementation of such cutting-edge ML technologies for the solar plant’s design, forecasting, maintenance, and control provides digital transformation of solar energy into smart solar energy. These ML technologies are implementations of a basic ML model class which is represented on Figure 11 as an UML class diagram.

Figure 11 shows a method “Add” of a basic ML model class. This method adds a quantum layer into a classical ML model to create a quantum ML model. This method can be implemented by an integrating framework (Pennylane) for quantum computer simulators [140]. A quantum-based solar plant failure detection model was developed in [141].

IoT (Figure 11) provides an optimal solution to collect solar energy big data wirelessly (Figure 11). In [137], the solution researchers integrated a solar plant failure detection ML model. Future research can comprise the elaboration of a pipeline for implementing a real time solar plant diagnostic system based on IoT, EC, and/or TinyML technologies [138]. In [139], researchers developed based on EC a lightweight ML real-time solar plant failure detection model. Recent cloud-based monitoring solutions were developed in [141,142]. Forthcoming ML technologies for solar energy will integrate cloud-based solutions in which these technologies take full benefits of ML parallelism, data parallelism, practically limitless big data and ML knowledge storage, and almost boundless parallel computational resources.

The most complex issue of a smart sensor system is the self-learning of a sensor system. The potential methods for smart sensor’s adaptive learning are memristors and a spiking neural network [143]. In the future, a smart solar plant system will integrate a self-supervised learning ML model zoo [144] that provides optimum ML technologies for the solar plant’s design, forecasting, maintenance, and control.

Within the EU COVID-19 strategic reply, the smart energy standards define a cloud platform specification for distributed solar big data ecosystem that will provide creation of effective ML technologies for smart solar energy. The open solar energy data sources, including big data, provide the development of cutting-edge ML technologies in solar energy. Therefore, more open datasets with real data from solar sensor systems should be shared with the research community.

The integration of the cloud, big data, ML, EC, IoT, quantum, and sensor technologies will provide high efficiency of ML technologies for the solar plant’s design, forecasting, maintenance, and control. Implementation of these technologies for the solar plant’s design, forecasting, maintenance, and control provides digital transformation of solar energy into smart solar energy. The integrated electric grids are becoming increasingly reliable and overall solar production costs are minimized.

## 5. Conclusions

We presented a structured (mostly in benchmark tables) review of the advances in ML technologies for the solar plant’s design, forecasting, maintenance, and control where most of the reviewed articles were published within the last five years. 

ML methods are key elements of smart sensor systems of solar plants because they automatically create smart models for the solar plant’s design, forecasting, maintenance, and control and more effectively analyze exponentially growing big data as compared to traditional methods. In this review, we briefly summarized our self-adaptive models for sizing, forecasting, maintenance, and control of a solar plant based on an MFNN that were automatically created with regard to a task’s complexity and overfitting problem.

In the reviewed studies, the smart sensors where signals of image sensor and the CNN blocks strongly correlate provide high performance. There is an argumentative direction to substitute non-NN smart models with a DNN-based model for the solar plant’s design, forecasting, maintenance, and control because DNN provides better information processing quality and performance as compared to non-NN smart models. The impact of the ML technologies based on the proposed implementation scheme on a solar plant value chain will mostly be associated with the cost of software development which implements a ML sensor system based on ONNX, a developed software’s integration with SCADA, and maintenance.

The most complex issue of a smart sensor system is the self-learning of a sensor system. The potential methods for adaptive sensor learning are memristors and a spiking neural network.

In addition, we have outlined several problems that can be considered for future research in field of smart solar energy:

- In forecasting and failure detection, the usage of the DNNs such as transformers based on an attention mechanism is a promising alternative.
- For failure detection and classification, there is a need to study the MPPT algorithms based on RL and a spiking neural network under failure conditions.
- For diagnosis of a solar plant system based on thermal images, the usage of GANs is a promising alternative.
- There is a need to propose a pipeline for implementing a real-time solar plant diagnostic system based on IoT, EC, and/or TinyML technologies.
- The development of ML algorithms for real-time processing and decision making are most in demand in solar plant systems.

The long-term contribution, including increased capacity of solar energy, depends on solving the remaining tasks of coupling to electric grids, high costs, and low efficiency, mainly through the research and development of a smart solar plant system based on the integration of cutting-edge technologies, including DNN. Within the EU COVID-19 strategic reply, the smart energy standards define a cloud platform specification for a distributed smart solar big data ecosystem that will provide the creation of effective ML technologies for smart solar energy. The open solar energy data sources, including big data, provide the development of cutting-edge ML technologies in solar energy. Therefore, more open datasets with real data from solar plant sensor systems should be shared with the research community. In order to achieve the smart optimization and high efficiency of solar energy, the cloud, big data, ML, EC, IoT, quantum, and sensor technologies need to be adaptively combined and implemented as smart grid, home, and city applications. The integration of the above-mentioned cutting-edge technologies will provide high efficiency of ML technologies for the solar plant’s design, forecasting, maintenance, and control. Implementation of these technologies for the solar plant’s design, forecasting, maintenance, and control will provide digital transformation of solar energy into smart solar energy. The integrated electric grids are becoming increasingly reliable, and overall solar production costs are minimized. Forthcoming ML technologies for solar energy will integrate cloud-based solutions, in which these technologies take full benefits of the ML parallelism, data parallelism, practically limitless big data and ML knowledge storage, and almost boundless parallel computational resources.

## Figures and Tables

**Table 3:** Comparison of meta-heuristic algorithms.

Open Dataset	Data Source Location	Description
Duke California Solar Array Dataset [27]	-	Over 400 km^2^ of imagery and 16,000 hand-labeled solar arrays
SOLETE [28]	City: Roskilde, Denmark. Latitude and longitude: 55.6867, 12.0985	Meteorological and active power 15 months dataset from PV array
Desert Knowledge Australia Center Dataset [29]	-	Data of solar technologies spanning multiple types, ages, models, and configurations
Girasol [30]	Albuquerque, USA	A meteorological (10 min sampling interval), insolation (a sampling rate ranging from 4 to 6 samples per second), and images (sampling interval of the cameras is 15 s) 242 days (of 3 years) dataset
ESOLMET-IER Dataset [31]	Institute of Renewable Energies UNAM, station “ESOLMET-IER”	Solar metric and meteorological dataset
The National Solar Radiation Data Base (NSRDB) [32]	The USA and neighboring countries	Solar insolation and meteorological 23 years dataset
Photovoltaic Thermal Images Dataset [33]	66 MW PV plant in Tomboruk	Thermal images of PV arrays with the presence of one or more anomaly cells and their respective masks
Pecan Street Dataset [34]	-	1300 customer loads one-year dataset

**Table 4:** Comparison of the parameter identification models.

Algorithm	Outperforms	Diode Model	RMSE
ANN [35]	RBF-NN	SDM	Low
ANN [36]	ANFIS	SDM	Low
Flexible PSO [37]	Classical PSO	SDM and DDM	High
Whale optimization [38]	Classical PSO	DDM	Moderate
Tree-growth-based optimization (TG) [39]	Two-step Linear Least-Squares (TSLLS) method, Reduced forms RF, Artificial bee swarm optimization (ABSO), Harmony search-based algorithm (HS), Particle swarm optimization (PSO) algorithm, Genetic algorithm (GA), analytical 5-point method (An.5-Pt), the Lambert W (LW) function, Newton method, Conductance method, and pattern search	SDM	High
Memetic adaptive differential evolution (MD) [40]	GA	SDM	Low
Artificial Bee Colony (ABC) [41]	Classical ABC	SDM and DDM	Low
JAYA-based [42,47]	Covariance matrix adaptation evolution strategy (CMAES), Grey Wolf Optimizer (GWO), Teaching-learning-based artificial bee colony (TLABC), Transactional agents for pervasive computing (TAPSO), ML-based stealing attack methodology (MLBSA), Generalized oppositional teaching learning-based optimization (GOTLBO)	SDM	Low
Chaos Game Optimization (CGO) [43]	W, TG, MD, applied chaotic reproduction optimization (CARO) [41], modified simplified swarm optimization algorithm (MSSO) [48], Cuckoo search algorithm (CSA) [49], Biogeography optimization algorithm-based heterogeneous cuckoo search (BBO-HCS) algorithm [50]	SDM	Low
Supply-Demand-Based [44]	Backtracking Search Algorithm, Grey Wolf Optimizer, Bernstein–Levy Search Differential Evolution Algorithm, Crow Search Optimizer, and Manta Ray Foraging Optimizer	TDM	Low

**Table 5:** Performance comparison of the parameter identification models for the 57 mm dia RTC France solar cell [45].

Single Diode Model		Sl. No.	Double Diode Model	
RMSE	Algorithm		Algorithm	RMSE
7.27 × 10^−4^	BPFPA [45]	1	BPFPA [45]	7.23 × 10^−4^
7.84 × 10^−4^	FPA [45]	2	FPA [45]	7.73 × 10^−4^
9.45 × 10^−4^	MPCOA [45]	3	MPCOA [45]	9.22 × 10^−4^
9.86 × 10^−4^	STLBO [45]	4	STLBO [45]	9.82 × 10^−4^
9.86 × 10^−4^	R-JADE [45]	5	R-JADE [45]	9.82 × 10^−4^
9.86 × 10^−4^	TVIWAC PSO [45]	6	ABC + NMS [45]	9.82 × 10^−4^
9.86 × 10^−4^	BMO [45]	7	TAPSO [42]	9.8269 × 10^−4^
9.86 × 10^−4^	ABC + NMS [45]	8	MLBSA [42]	9.8285 × 10^−4^
9.86 × 10^−4^	ABC [45]	9	PGJAYA [42]	9.8298 × 10^−4^
9.86 × 10^−4^	BBO-M [45]	10	GOTLBO [42]	9.8299 × 10^−4^
9.86 × 10^−4^	LM + SA [45]	11	BMO [45]	9.83 × 10^−4^
9.8602 × 10^−4^	TLABC [42]	12	BB0-M [45]	9.83 × 10^−4^
9.8602 × 10^−4^	TAPSO [42]	13	ABSO [45]	9.83 × 10^−4^
9.8602 × 10^−4^	MLBSA [42]	14	TLABC [42]	9.8407 × 10^−4^
9.8602 × 10^−4^	GOTLBO [42]	15	ABC [45]	9.86 × 10^−4^
9.8602 × 10^−4^	PGJAYA [42]	16	IGHS [45]	9.86 × 10^−4^
9.8602 × 10^−4^	HAJAYADE [42]	17	IJAYA [42]	9.8631 × 10^−4^
9.860219 × 10^−4^	CGO [43]	18	JAYA [47]	9.8934 × 10^−4^
9.86022 × 10^−4^	BBO-HC [50]	19	CMAES [42]	9.9015 × 10^−4^
9.86023 × 10^−4^	CSA [49]	20	CLPSO [47]	9.9894 × 10^−4^
9.8605 × 10^−4^	CMM-DE/BBO [47]	21	CMM-DE/BBO [45]	1.0088 × 10^−3^
9.8607 × 10^−4^	MSSO [48]	22	DE/BBO [47]	1.0255 × 10^−3^
9.8625 × 10^−4^	IJAYA [42]	23	BLPSO [47]	1.0628 × 10^−3^
9.8665 × 10^−4^	CARO [41]	24	GGHS [45]	1.07 × 10^−3^
9.87 × 10^−4^	PSA [45]	25	GWO [42]	1.1429 × 10^−3^
9.89 × 10^−4^	IADE [45]	26	HS [45]	1.26 × 10^−3^
9.8946 × 10^−4^	JAYA [47]	27	SA [45]	N. S
9.91 × 10^−4^	GGHS [45]	28	PSO [45]	N. S
9.91 × 10^−4^	ABSO [45]	29		
9.93 × 10^−4^	IGHS [45]	30		
9.95 × 10^−4^	HS [45]	31		
9.9633 × 10^−4^	CLPSO [47]	32		
9.9922 × 10^−4^	DE/BBO [47]	33		
1.0023 × 10^−3^	GWO [42]	34		
1.0272 × 10^−3^	BLPSO [47]	35		
1.70 × 10^−3^	SA [45]	36		

**Table 6:** Comparison of ML sizing methods.

Sizing Method	Dataset	Performance	Contribution
Generalized RNN [51]	Meteorological and load demand dataset from five Malaysian sites	MAE% is 0.6%	-
CNN [52]	Duke California Solar Array dataset [23]	Object-based performance metric is 0.76	CNN creates semantic segmentation SolarMapper [53]
DNN framework [54]	Behind-the-meter load dataset that includes erroneous and mislabeled training data	MAE% in estimation of a PV tilt and azimuth values are 10.1% and 2.8%, correspondingly	-
MFNN [5]	Two-year dataset of total insolation, meteorological parameters which was collected at the site of Abakan	MAE% is 0.6% which is superior to PSO	Automatic creation, self-adaptation MFNN based on the authors’ software [20]
ML optimization method based on ANN and heuristic optimizers [55]	One-month datasets of meteorological parameters which were collected at the different climatic China regions	The annual equivalent overall output energy increased by 4.48% as compared to a Taguchi standard orthogonal array	Within the application of smart cities researchers design a renewable system that includes solar-to-electricity conversion.

**Table 10:** Comparison of ML Technologies for PV Diagnostics.

ML Method	Localize/Identify Failure	Performance	Dataset
YOLOv4 [106]	Light reflex,	0.96	Preprocessed dataset of thermographic images
	hot spot,	0.956	
	short circuit,	0.905	
	faulty string/sunbstring,	0.969	
	“good” module	0.997	
CNN [107]	Binary classification of hot spots	Average performance on test dataset is 98%, a range of processing speed is [0.001, 2 min]	Preprocessed dataset of thermographic images
Hybrid mask region CNN [108]	classify three failures: one damaged cell, nonadjacent, and adjacent damaged cells	RMSE of 26.85 W/m^2^, 19.78 W/m^2^ and 39.2 W/m^2^ for PC, MC, and TF solar plants correspondingly	Dataset of thermal images generated by infrared sensors installed in a UAV
Modified VGG16 [109]	detect a failure (bird’s drops over a PV array)	Average performance on test dataset is 93%	Dataset of 1000 affected images
SVM, naive Bayes, kNN, DT, RF and pre-trained DNN [110]	Delamination, hot spot, glass damages, decolorization, and snail trails	Best accuracy is 100%	Dataset of aerial images.
DIP filters and SVM classifier [111]	Classification into 10 different classes (1 healthy and 9 failure modes including warm module/substrings/cells, hot spot, etc.)	The average accuracy on test dataset is 94.4%	The thermographic images dataset that includes 16,000 samples (1600 for each class)
VGG16 [112]	Localization and classification into 6 different classes (1 healthy and 5 failure modes including overheated module/substrings hot spot, etc.)	The mean F1-score is 94.52%	Dataset of thermal infrared images was collected from 28 solar plants, which have 93220 solar panels

## Data Availability

Not applicable.

## Abbreviations

SCADA	Supervisory control and data acquisition
ML	Machine learning
PID	Proportional integral derivative
CRISP DM	Cross Industry Standard Process for Data Mining
ONNX	Open neural network exchange
PCA	Principal component analysis
NN	Neural network
LR	Linear regression
SVM	Support vector machine
RF	Random forest
DT	Decision tree
DL/DNN	Deep neural learning/network
ANN	Artificial neural network
RNN	Recurrent neural networks
CNN	Convolutional neural networks
XGBoost	Extreme gradient boosting
SGD	Stochastic gradient descent
ACC	Accuracy
MCC	Matthew’s correlation coefficient
ROC	Receiver operating characteristic
AUC	Area under the curve
MAE	Mean absolute error
CEEMDA	Complete ensemble empirical mode decomposition with adaptive noise
nRMSE	Normalized RMSE
nMAE	Normalized MAE
STLBO	Simplified Teaching Learning Based Optimization
ANFIS	Adaptive network based fuzzy inference system
PSO	Particle swarm optimization
QK-CNN	quad-kernel deep CNN
MPPT	Maximum power point tracking
NSRDB	National solar radiation data base
MPP	Maximum power point
CGO	Chaos game optimizer
CARO	Applied chaotic reproduction optimization
RH	Relative humidity
CSA	Cuckoo search algorithm
BMO	Bird Mating Optimization algorithm
MSSO	Modified simplified swarm optimization algorithm
CI	Cloud index
WS	Wind speed
Pr	pressure
MD QPSO	Multidimensional quantum behaved particle swarm optimization
C-LSTM	Constrained LSTM
kNN	k-Nearest Neighbors
ETR	Extra trees regressor
RMSE	Root-mean square error
CWT	Continuous wavelet transform
IFR	Infrared
UAV	Unmanned Aerial Vehicle
IS	Isolation Forest
LOF	Local Outlier Factor
STC	Standard test conditions
DQL	Deep Q-learning
DDPG	deep deterministic policy gradient
RBF NN	radial basis function neural network
TCT	Total-cross-tied
SD-PAR	Shade Dispersion Physical Array Relocation
MHHO	Modified Harris Hawks Optimizer
BPFPA	Bee Pollinated Flower Pollination Algorithm
FPA	Flower Pollination Algorithm
MPCOA	Mutative Scale Parallel Chaos Optimization Algorithm
MFNN	Modified fuzzy neural net
R-JADE	Repaired adaptive differential evolution
TVIWAC PSO	PSO with time varying inertia weight and acceleration coefficients
BBO-HCS	Biogeography optimization algorithm based heterogeneous cuckoo search
ABC	Artificial Bee Colony
NMS	Nelder Mead algorithm
BBO	Biogeography Based Optimization
LM	Levenberg–Marquardt
PSA	Parallel Swarm Algorithm
IADE	Improved Adaptive Differential Evolution
GGHS	Grouping based global harmony search
ABSO	Artificial Bee Swarm Optimization
IGHS	Innovative Global Harmony Search
HS	Harmony Search
SA	Simulated Annealing
